# Effect of Arabica Coffee Husk on Plasma Lipids and Obesity on Albino Rats

**DOI:** 10.1002/fsn3.71987

**Published:** 2026-06-04

**Authors:** Shaheed Mohammed Alshaikhsaleh

**Affiliations:** ^1^ Department of Food and Nutrition Sciences, College of Agriculture and Food Sciences King Faisal University Al‐Ahsa Saudi Arabia

**Keywords:** adipose tissue, coffee husks, hyperlipidemia, polyphenols

## Abstract

Hyperlipidemia is a major risk factor for the development of cardiovascular diseases. This study investigated the effects of coffee husk (CH) on lipid profile and plasma glucose levels in rats over an 8‐week period. Eighteen male Albino rats were divided into three groups: a control group received a standard diet, a high‐fat diet (HFD) group received a standard diet containing 20% palm olein oil, and a CH group received a standard diet containing 20% palm olein oil and 7% CH. Different types of white adipose tissues were examined by hematoxylin and eosin staining. Results demonstrated that the control group presented the lowest levels of total cholesterol (TC), total triglyceride (TG), low‐density lipoprotein (LDL‐C), and plasma glucose. The HFD group showed significantly increased levels of all parameters compared to both control and CH groups (*p* < 0.05). The CH group recorded moderate levels for all measured indicators, which may reflect the positive effect of the husks on blood lipids. Moreover, the size of adipose tissue cells was smaller in the CH group compared to the HFD group. HPLC results illustrated that CH contains 4‐hydroxybenzoic acid, rosmarinic acid, quercetin, caffeic acid, catechin hydrate, and gallic acid (254.2, 5.415, 4.471, 3.381, 2.865, and 2.823 ppm, respectively). The positive effect of CH could be attributed to the presence of 4‐hydroxybenzoic acid and rosmarinic acid. In conclusion, coffee husk could potentially be used as a supplement to lower plasma lipid levels. Further research should be conducted to examine the coffee husks' impact on chronic diseases.

## Graphical abstract

Feeding on a high‐fat diet supplemented with Arabica coffee husks (CH) for 8 weeks led to an improvement in plasma lipids and adipose tissues in rats compared to the high‐fat diet.

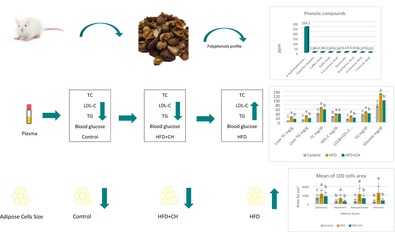

## 1 Introduction

Atherosclerosis is one of the main cardiovascular diseases that leads to the cause of death in developed countries (Li et al. 2021). Increased levels of total plasma cholesterol, especially LDL‐C, can lead to cardiovascular diseases, which are the leading cause of death (Kruit et al. 2006). A diet rich in fats is associated with obesity and elevated plasma lipid levels, especially LDL‐C (Ozen et al. 2022). The obesity rate is increasing worldwide, and the impact of obesity control strategies on the obesity rate is low due to the complex pathogenesis. The type and amount of fat consumed are considered major factors in the causes of obesity (Gao et al. 2024).

Many treatments have been developed to lower LDL‐C; for instance, statins are the first‐line treatment for lowering LDL‐C and can reduce it by about 60%, and they also lower the TG level (Feingold 2000). One of the side effects of prolonged use of statins is the appearance of symptoms in the muscles (Ward et al. 2019). Allopathic treatments are currently the most commonly used for individuals with hyperlipidemia, but their problem lies in side effects and incompatibility with some patients. Therefore, it is necessary to resort to natural alternatives, especially from plant sources, which are referred to as functional foods (Gong et al. 2020). Recently, there has been significant interest in polyphenols for their potential therapeutic effects in disease prevention. Polyphenols are phytochemicals that contain phenolic rings and are primarily found in vegetables, fruits, grains, and nuts (Sun et al. 2021). Polyphenols in pomegranate peels have a positive effect in treating hyperlipidemia and also have a significant impact on increasing the expressions of mRNA for LXRα (Liver X receptor alpha) and the target genes ABCA1 (ATP‐binding cassette transporter A1), which play an important role in regulating cholesterol flow to cells (Zhao et al. 2014). Zhucha is an ancient Uyghur medicine made from bamboo leaves and green tea. The effect of a mixture of flavonoids in bamboo leaves and phenols in green tea was compared to lipid‐lowering drugs and applied to rats with hyperlipidemia. The results showed that 75 and 500 mg/kg daily produced results similar to the lipid‐lowering drugs (Yang et al. 2015).

The biggest producers in the world of coffee fruit ( *Coffea arabica* L.) are Brazil, Vietnam, Colombia, and Indonesia (Cangussu et al. 2021). Coffee husks are the byproduct of the coffee bean production process and constitute about 30%–40% of the total coffee fruit (Azmi Afriansyah et al. 2023). Annually, around 2 billion tons of solid waste are produced during coffee production, with about 10 million tons of it being coffee husks. Most of these husks end up in landfills (Ali and Bhowmik 2025). Coffee is processed after harvesting through solar drying, followed by husking to obtain green coffee. The coffee fruit consists of coffee surrounded by the silver skin, then the parchment, then the mucilage, then the pulp, and finally the outer husk (Ali and Bhowmik 2025). The chemical composition of coffee husks, the predominant component is dietary fiber at 65.83%, while the protein content is 10.03%, moisture 5.50%, ash 5.20%, and fat 5.01% (Cangussu et al. 2021). Coffee husks contain phenolic compounds such as chlorogenic acid, caffeic acid, quercetin, gallic acid, syringic acid, and ferulic acid (Abreu et al. 2024). The phenolic compounds present in coffee husks exhibit inhibitory activity with ABTS (96.4%), DPPH (75.1%), and FRAP (71.2%) (Silva et al. 2024).

In a previous study on coffee husks, rats fed HFD supplemented with 15% coffee husks showed a significant decrease in total cholesterol, LDL‐C, and VLDL‐C, and an increase in HDL‐C compared to the control group HFD only (El‐din and Elsawy 2021). Feeding on a high‐fat diet supplemented with the silver skin of the coffee led to a reduced rate of weight gain, a decrease in white fat mass, and a reduction in alanine aminotransferase, aspartate aminotransferase, and alkaline phosphatase activities (El‐Anany and Ali 2018). In a study to evaluate the ability of Arabica and Robusta coffee leaf extracts to reduce the digestion and absorption of fats in the gastrointestinal tract, the extracts showed an inhibitory effect on pancreatic lipase and a decrease in the solubility of micellar cholesterol and bile acid binding. The study recommended that leaf extracts may be useful in preventing hyperlipidemia (Sansri et al. 2024). Consuming 3–4 cups of coffee daily may reduce the risk of developing type 2 diabetes and obesity (Santos and Lima 2016). In a study simulating digestion to evaluate the ability of coffee pulp to reduce fat emulsification and fat digestion, as well as its capacity to lower lipids in HepG2 cells after promoting fat accumulation within the cells, the study indicated that the phenolic compounds present in coffee pulp may be responsible for binding bile acids and inhibiting the activity of the lipase and the HMGCR receptor (Braojos et al. 2024). Yogurt enhanced with coffee pulp has the ability to improve blood lipids, especially reducing blood cholesterol and decreasing visceral fat, and it did not affect liver and kidney functions in hypercholesterolemic experimental rats compared to the control group (Zainal Abidin et al. 2025).

A review study indicates that caffeine, chlorogenic acid, and diterpenes are considered active compounds in coffee that may exhibit anti‐adipogenic, anti‐obesity, and anti‐diabetic effects (Sirotkin and Kolesárová 2021). However, some study results may show inconsistent and varied outcomes, which could be attributed to the addition of cream or sugar (Kusumah and Gonzalez de Mejia 2022).

Therefore, this study aims to evaluate the effect of Arabica coffee husks on blood plasma lipids (total cholesterol, high‐density lipoproteins, low‐density lipoproteins, and triglycerides) and also the effect of the husks on white adipose tissues (epididymal, mesenteric, retroperitoneal, and perirenal), and to correlate the results with the phenolic compounds present in coffee husks.

## 2 Material and Method

The coffee husks were purchased from a local market in the city of Jazan in the southern region of the Kingdom of Saudi Arabia. These husks are from Arabica coffee, locally known as Khawlani coffee, which is grown in high‐altitude areas of approximately 1200–2000 m above sea level. Total cholesterol (TC), total triglyceride (TG), blood glucose, and high‐density lipoprotein (HDL‐C) kits were purchased from Quimica Clinica Aplicada, Spain.

### 2.1 Coffee Husk Powder Preparation

The coffee husks were ground in a grinder (HomeLec, HCG‐500, China) at 34,000 rpm, and then the ground material was sifted using a sieve to obtain a powder with a particle size of ≤ 300 μm.

### 2.2 Proximate Composition of Coffee Husk

The total nitrogen by the Kjeldahl method was determined by Kirk and Sawyer (1991), and the protein content was calculated using 6.25 as a factor. One gram of the ground coffee husk sample was weighed in a digestion tube, then digestion tablets (potassium sulfate and copper) were added, followed by five granules (glass balls), and finally 25 mL of concentrated sulfuric acid. The tube is transferred to the digestion unit (BUCHI K‐425, Switzerland) at a temperature of 400°C for one and a half hours (until its color becomes transparent). The sample is distilled using the distillation unit (BUCHI K‐370, Switzerland) by adding distilled water to dilute the sample, then adding sodium hydroxide until the foaming stops, and then running the distillation unit. Ammonia gas is received in a flask containing 50 mL of 4% boric acid with a methylene blue indicator. The color of boric acid turns green when exposed to ammonia gas. The solution (ionized boric acid) is titrated with 0.01 M sulfuric acid until the endpoint (dark gray color). The percentage of crude protein is calculated using the following equation:

$$\text{Percentage of crude protein}=\left(\left(14\times 0.01\times V\times 6.25\right)/\left(W\times 1000\right)\right)\times 100$$

where *V* is the volume (mL) of sulfuric acid, and *W* is the weight of the sample (g).

Moisture was estimated by oven‐drying at 105°C (Nielsen 2019). Three grams of a ground coffee husk sample were weighed in a metal dish of known weight and then placed in a drying oven (Binder GmbH Bergstr, Tuttlingen, Germany) at 105°C for 8 h. After drying, the sample with the metal dish was weighed, and the moisture content was calculated using the following equation:

$$\text{Percentage of moisture}=\left[\left(\left(\text{W1}+\text{W2}\right)-W3\right)/\text{W2}\right]\times 100$$

where W1 is the metal dish weight, W2 is the sample weight, and W3 is the sample with the metal dish after drying.

Crude fat was estimated by solvent extraction (Nielsen 2019). Three to five grams of the sample were weighed in the extraction thimble, and the empty extraction flask was weighed. Then, a Soxhlet extraction system was assembled and operated for 16 h, after which the solvent was removed from the extraction flask, leaving only the fat. The extraction flask with the fat is weighed, and then the fat percentage is calculated using the following equation:

$$\text{Percentage of crude}\;\mathrm{fat}=\left(\left(\text{W3}-W2\right)/\text{W1}\right)\times 100$$

where W1 is the sample weight, W2 is the extraction flask weight, and W3 is the weight of the extraction flask and fat.

The ash was estimated by incinerating the sample (Nielsen 2019). Three grams of the sample were weighed into a crucible of known weight, then the crucible was placed in a muffle furnace (Thermolyne 6000, Thermo Fisher Scientific) at a temperature of 225°C (initial burn). The temperature was then raised to 550°C for 8 h until ash was formed.

$$\text{Percentage of}\;\mathrm{ash}=\left(\left(\text{W3}-W2\right)/\text{W1}\right)\times 100$$

where W1 is the sample weight, W2 is the crucible weight, and W3 is the weight of the crucible and ash.

The crude fiber was estimated using the method described in Nielsen (2019). Three grams of the sample were weighed in a crucible, then the sample was soaked in acetone for 10 min, and this was repeated twice, followed by rinsing the sample with distilled water. The crucible was placed in FibertecTm8000 (Foss, Denmark) and treated with 1.25% H_2_SO_4_ for one and a half hours with heating, then rinsed with distilled water and treated with 1.25% NaOH for one and a half hours with heating, then rinsed with distilled water. The sample is dried in a drying oven at 105°C for 2 h, then the crucible is weighed. The sample is ashed, then the crucible is weighed, and the percentage of crude fiber is calculated using the following equation:

$$\text{Percentage of crude fiber}=\left(\left(\text{W2}-W3\right)/\text{W1}\right)\times 100$$

where W1 is the sample weight, W2 is the crucible weight after drying, and W3 is the weight of the crucible and ash.

### 2.3 Extract Preparation

Thirty‐six grams of the coffee husk powder were added to a clean flask, and 180 mL of 80% ethanol was added to the flask. The flask contents were homogenized and transferred to a shaking water bath and kept at 25°C for 8 h. Then the flask content was filtered using paper filter No. 1, and the solution was evaporated using a rotary evaporator (BUCHI R‐210, Switzerland). The extract yield was calculated by weighing the flask before and after evaporating (Bouhlali et al. 2017).

### 2.4 Antioxidant Activity

The IC50 test was conducted to determine the amount of husk extract needed to inhibit 50% of the DPPH. The DPPH radical scavenging assay was performed according to the method described by Al Qaisi et al. (2024) with slight modification. One mL of different concentrations (1, 5, 10, 100, and 500 μg/mL) of the sample extract and vitamin C (as a standard) was added to 3 mL of ethanolic DPPH solution (4 mg of DPPH in 100 mL ethanol). Then the tubes were incubated in a dark place at room temperature for 30 min. The absorbance for the tubes was taken at 517 nm using a spectrophotometer (UV‐1800 Shimadzu, Japan). A blank sample was prepared using ethanol.

### 2.5 Phenolic Compound Profile

The phenolic compounds of the coffee husk were analyzed by HPLC. The instrument brand model is Agilent Technologies. The column was an LC‐18 Intersil ODS‐3 (4.6 × 250 mm, 5 μm particle size) by GL Sciences, Tokyo and the column temperature was 25°C. The flow rate was 0.8 mL/min, and the mobile phase was a mixture of 3% acetic acid (A) and methanol (B). Twenty microliters of the samples were injected into the mobile phase by the following gradient: 80% A and 20% B as initial condition, 70% A and 30% B after 25 min from the initial, 67% A and 33% B after 60 min, 50% A and 50% B after 70 min, 30% A and 70% B after 72 min, 20% A and 80% B after 75 min, 7% A and 93% B after 77 min, 0% A and 100% B after 79 min, 50% A and 50% B after 80 min, 93% A and 7% B after 85 min until 90 min. The detector was from Agilent Technologies, 1260 Infinity model.

### 2.6 Biological Experiment

The biological experiment was conducted at the College of Agricultural and Food Sciences at King Faisal University in October 2024. Eighteen male Albino rats, 4 weeks old and weighing between 80 and 100 g, were purchased from the animal house and divided into three equal groups. In a cage made of polypropylene with an area of 821 cm^2^ (26 cm in height, 43 cm in length, and 20.1 cm in width), six rats were placed randomly as one group. The floor of the cage contains sawdust that is replaced once a week. These rats were then fed a standard diet for a week to acclimate them. The standard diet consists of 24.3% protein, 4.7% fat, 40.2% carbohydrates, 4% fiber, and 7.6% ash, with an energy density of 3 kcal per gram (Teklad rodent diet 8604). Subsequently, one group was fed the standard diet (control group), one group was fed a standard diet containing 20% w/w vegetable oil (palm olein oil) as a high‐fat diet (HFD) group and another group (HFD + CH) was fed a standard diet containing 20% w/w vegetable oil (palm olein) along with ground coffee husks at a ratio of 7% w/w dry matter basis for 8 weeks with open access to food and water at a temperature of 20°C and 40% humidity, in a lab with 12 h of light and 12 h of darkness. The weight of the rats was measured in the first week and at the end of the experiment (eighth week) to calculate the body mass index (BMI) and the percentage of body weight gain (%BWG). Additionally, the amount of feed consumed was measured in the first and eighth week by weighing the food before presenting it to the rats and then weighing the remaining food at the same time the next day. This was done daily for a week. Blood samples were taken from the rats in the first week before feeding them the different diets and at the end of the eighth week.

### 2.7 Plasma Collection

Blood samples were taken, and plasma was obtained using the method (Alshaikhsaleh and Asiri 2025). After fasting the rats for 8 h, the rats were anesthetized by diethyl ether for 2–3 min (until they lose consciousness). Then, the blood sample was drawn from the eye vein using a sodium‐heparinized capillary tube (75 mm long and 1.1–1.2 mm internal diameter from Marienfeld, Germany). The blood sample was received in a 1.5 mL Eppendorf tube containing EDTA, and the sample was mixed. And after all the samples have been collected, they were centrifuged at 3000 rpm (Hermle Labortechnik GmbH, Z 233M‐2, Germany) for 18 min to obtain the blood plasma. After that, the following analyses are performed on the obtained blood plasma: total cholesterol (TC), high‐density lipoproteins (HDL‐C), total triglycerides (TG), and blood glucose. Low and very low‐density lipoprotein VLDL‐C and atherosclerosis index were also calculated using the following formulas (Alshaikhsaleh et al. 2025):

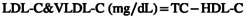




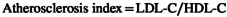




At the end of the eighth week, the rats were dissected, and the heart, kidneys, spleen, and liver were removed and weighed. A part of the liver was kept in a saline solution (0.09% sodium chloride) for liver TC and TG tests as described in Abd El‐Gawad et al. (2005). Additionally, the white adipose tissues (epididymal, mesenteric, retroperitoneal, and perirenal) were removed, weighed and preserved in formalin solution for histopathological analysis.

### 2.8 Histological Analysis

The white adipose tissues were processed using a tissue processor (Sakura Finetek 4640‐B, USA) by immersing the tissue in six consecutive jars containing 70% ethanol, 80% ethanol, 90% ethanol, 100% ethanol, xylene, and finally molten paraffin wax (63°C). After processing, the tissues were cast in molds using paraffin wax (MPS P1, SLEE Medical GmbH, Germany), then sectioned to a thickness of 4 μm using a (Leica Microsystems RM2235, Germany). The sections were then stained with hematoxylin and eosin. Finally, the slides were examined using a light microscope equipped with a camera at 200×, and images of the sections were taken using an (Olympus BX51 photomicroscope). The area of 100 cells from the same region was measured without any bias (measuring the area of 100 adjacent adipocytes) for each adipose tissue in each group (Karam et al. 2018; Moon et al. 2019).

### 2.9 Statistical Analysis

All the data were analyzed using SAS 9.0 software (SAS Institute). The data were expressed as means ± standard deviation, and the data were analyzed using a complete randomized design one‐way ANOVA. The differences between means were tested using the Tukey test to assess significant differences; a *p*‐Value of less than 0.05 was considered statistically significant. All assumptions for the ANOVA were verified prior to analysis, including normality and homogeneity of variance using the Shapiro–Wilk test and Levene's test, respectively. The Welch's ANOVA was used to compare (LDL‐C&VLDL‐C, Atherosclerosis index, BMI, and Liver TG) levels because the assumption of homogeneity of variances was violated.

## 3 Results

Table 1 shows the results of the chemical analysis of coffee husks. It found that the husks have very low moisture content, which may be due to the drying processes of coffee cherries before the husks are removed as a preliminary step in coffee processing. There is a good percentage of protein in coffee husks, around 6.75%, and it contains a small amount of crude fat. While the crude fiber is the highest percentage in the husks. The extract percentage in the husks was about 24%.

**TABLE 1:** Nutritional and phytochemical profile.

Parameter	Content in %
Moisture	4.01 ± 0.28
Protein	6.75 ± 0.01
Crude fat	3.73 ± 0.40
Ash	6.34 ± 0.11
Crude fiber	22.68 ± 1.69
Dry Extract	23.74 ± 0.48

Phenolic compound	ppm
3‐Hydroxybenzoic acid	< LOQ
4‐Hydroxybenzoic acid	254.2
Benzoic acid	< LOQ
Catechin hydrate	2.865
Chlorogenic acid	< LOQ
Caffeic acid	3.381
Epicatechin	< LOQ
Gallic acid	2.823
Hesperidin	< LOQ
P‐Coumaric acid	0.367
Quercetin	4.471
Rosmarinic acid	5.415
Sinnapic acid	< LOQ
Syringic acid	< LOQ
t‐Cinnamic acid	0.377
t‐Ferrulic acid	0.011

*Note:* Percentage of moisture, protein, crude fat, ash, crude fiber, and dry extract are shown as mean ± SD on *n* = 3 replicate per sample.

Abbreviation: LOQ, limit of quantification.

**FIGURE 1:**
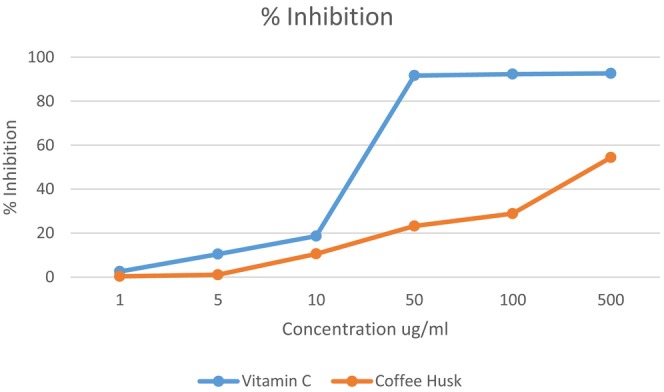
Evaluation of IC_50_ of coffee husk extract and ascorbic acid.

As for the phenolic compounds in coffee husks, the predominant compound was 4‐Hydroxy benzoic Acid with a content of 254.2 ppm, followed by Rosmarinic Acid, then Quercetin, then Caffeic Acid, then Catechin Hydrate, and finally Gallic Acid (5.415, 4.471, 3.381, 2.865, 2.823 ppm respectively). In a tutorial review study, they found that coffee husks contain 1.17–1.20 μg/g quercetin, 5.24–33.7 μg/g caffeic acid, and 3.47–30.37 μg/g gallic acid (Ali and Bhowmik 2025). As shown in Figure 1, the coffee husk extract exhibited DPPH radical scavenging activity, with an IC₅₀ value of 421 μg/mL.

Table 2 shows the results of plasma blood indicators, which are TC, HDL‐C, LDL‐C and vLDL‐C, atherosclerosis index, TG, blood glucose, and AST and ALT enzymes.

**TABLE 2:** Effect of feed on HFD and HFD with coffee husk on plasma parameters.

Parameter	Beginning	Group		
		Control	HFD	HFD+CH
TC (mg/dL)	41.24 ± 1.91	38.31 ± 3.14^c^	66.45 ± 1.39^a^	56.78 ± 1.20^b^
HDL (mg/dL)	27.20 ± 2.86	25.34 ± 1.33^b^	37.69 ± 1.14^a^	36.87 ± 1.14^a^
LDL & VLDL (mg/dL)	14.03 ± 1.96	12.96 ± 2.27^c^	28.76 ± 0.69^a^	19.91 ± 0.56^b^
Atherosclerosis index	0.52 ± 0.11	0.51 ± 0.08^b^	0.76 ± 0.02^a^	0.54 ± 0.02^b^
TG (mg/dL)	32.40 ± 3.78	32.32 ± 4.63^c^	47.66 ± 1.08^a^	39.48 ± 0.71^b^
Glucose (mg/dL)	79.24 ± 5.46	75.36 ± 7.21^c^	129.37 ± 1.00^a^	97.83 ± 1.09^b^
ALT (U/L)	22.03 ± 3.11	23.51 ± 0.11^c^	24.02 ± 0.09^a^	23.79 ± 0.08^b^
AST (U/L)	33.79 ± 2.49	33.50 ± 0.11^c^	34.01 ± 0.11^a^	33.77 ± 0.09^b^

*Note:* Data are shown as mean ± SD on *n* = 6 replicate per group. Values in the same row with different latter are significant different, *p* < 0.05. HFD, High fat diet; HFD + CH, High fat diet + coffee husks.

Regarding the total blood cholesterol levels, an increase was observed in the HFD group compared to the other groups. This reflects the effect of the high‐fat diet in increasing blood cholesterol levels. In contrast, TC levels in the HFD + CH group decreased by approximately 15% compared to the HFD group, which may indicate the positive effect of CH on total cholesterol levels. The normal range for total cholesterol levels in the blood of experimental rats is 10–54 mg/dL (Harini and Astirin 1970). This indicates that the control group had normal TC levels, while the HFD group was above the range, and the HFD + CH group was approximately 2 mg/dL higher than the normal level.

The high‐density lipoprotein levels in rats ranged from 21 to 54 mg/dL (Abd El Malik 2019). All groups were within the normal levels of HDL‐C. The HFD and HFD + CH groups had the highest HDL‐C levels with significant differences compared to the control group. This increase may be due to the elevated TC in the same two groups, which will affect the blood lipoproteins. As for LDL‐C, the levels were the lowest in the control group with significant differences compared to other groups, and the highest levels were in the HFD group. The results of the statistical analysis showed highly significant differences between all groups in LDL‐C according to the Welch test (F (2,8.98) = 554.92, *p* = < 0.0001). There were no significant differences between the control group and the HFD + CH group in the atherosclerosis index, as determined by the Welch test (F (1,5.77) = 0.11, *p* = 0.75), while the HFD group had the highest index with significant differences compared to the other groups (F (2,8.89) = 26.26, *p* = 0.0002). This may indicate the effect of CH on reducing the progression of atherosclerosis.

The total triglycerides in the blood of experimental rats are 26–145 mg/dL (Mahdi et al. 2020). The TG levels in rats fed different diets are within the normal range for rats. But the HFD group was the highest compared to all the groups with significant differences at 0.05. The lowest levels were in the control group, while the group fed a high‐fat diet supplemented with CH had blood TG levels that were intermediate between the control group and the HFD group.

Blood glucose levels in the control group were the lowest whereas the group fed a HFD had the highest blood glucose levels. As for the HFD + CH group, blood glucose levels were intermediate among the other groups. This may indicate the potential ability of CH to lower blood glucose levels in high‐fat diets or maintain the blood glucose levels. However, all groups in this study had blood glucose levels within the normal range for experimental rats, 50–135 mg/dL (Hidayaturrahmah et al. 2020).

As for AST and ALT, all treatments had similar values, while the statistical analysis showed significant differences between the treatments.

Table 3 shows the changes in body weight, %BWG, BMI, white adipose tissue, cholesterol, and triglycerides in the liver of rats fed different diets.

**TABLE 3:** Effect of feed on HFD and HFD with coffee husk on food intake, body weight, BMI, %BWG, origins weight, adipose tissues, and liver TC and TG.

Parameter	Group		
	Control	HFD	HFD + CH
Food intake beginning (g/day)	13.77 ± 0.14^a^	13.86 ± 0.09^a^	13.79 ± 0.09^a^
Food intake after 8 weeks (g/day)	16.28 ± 0.22^a^	16.24 ± 0.08^a^	16.20 ± 0.16^a^
Initial weight (g)	94.93 ± 4.22^a^	94.73 ± 4.90^a^	94.69 ± 4.00^a^
Final weight (g)	266.47 ± 5.26^c^	320.37 ± 4.57^a^	293.27 ± 3.64^b^
BMI (g/cm^2^)	0.58 ± 0.04^a^	0.57 ± 0.43^a^	0.58 ± 0.02^a^
%BWG	181.10 ± 11.35^c^	238.84 ± 15.34^a^	210.10 ± 10.82^b^
Heart (g)	1.87 ± 0.05^c^	2.27 ± 0.03^a^	2.07 ± 0.03^b^
Liver (g)	8.71 ± 0.67^c^	10.78 ± 0.35^a^	9.39 ± 0.58^b^
Kidney (g)	2.76 ± 0.31^b^	3.69 ± 0.36^a^	3.08 ± 0.40^b^
Spleen (g)	0.83 ± 0.05^c^	1.04 ± 0.05^a^	0.91 ± 0.05^b^
% Total adipose tissues	1.55 ± 0.02^c^	1.95 ± 0.02^a^	1.59 ± 0.02^b^
% Epididymal	0.40 ± 0.01^c^	0.52 ± 0.01^a^	0.45 ± 0.01^b^
% Mesenteric	0.74 ± 0.01^b^	0.82 ± 0.01^a^	0.72 ± 0.01^c^
% Retroperitoneal	0.33 ± 0.01^b^	0.51 ± 0.01^a^	0.32 ± 0.01^c^
% Perirenal	0.08 ± 0.00^b^	0.10 ± 0.01^a^	0.10 ± 0.01^a^
Liver TC (mg/g)	7.17 ± 0.07^c^	25.22 ± 0.75^a^	14.85 ± 0.17^b^
Liver TG (mg/g)	11.44 ± 0.15^c^	29.53 ± 0.92^a^	17.94 ± 0.53^b^

*Note:* Data are shown as mean ± SD on *n* = 6 replicate per group. Values in the same row with different small letter are significant different between groups, *p* < 0.05. HFD, High fat diet; HFD + CH, High fat diet + coffee husks.

The study results showed that the high‐fat diet led to significant physiological changes compared to the control group, while treatment with CH contributed to alleviating some of the negative effects of the HFD. The amount of food consumed at the beginning of the experiment and even after 8 weeks did not show any significant differences between the groups, indicating that the changes in weight and adipose tissue were not due to differences in the amount of food consumed but may be a result of metabolic effects.

**FIGURE 2:**
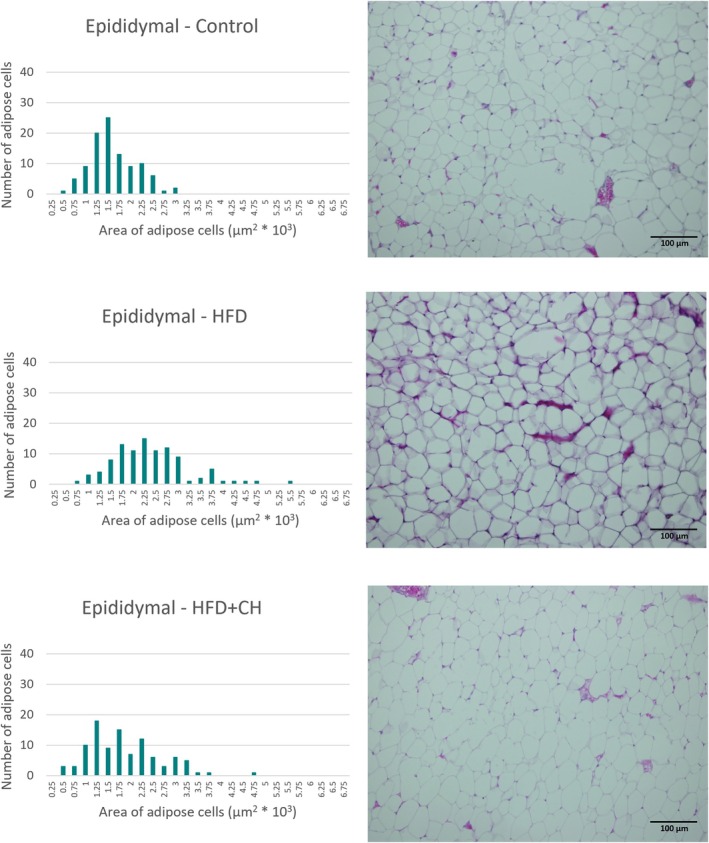
The microscopic image of adipose tissue (epididymal) and the cell area of 100 cells in the different groups. HFD, High‐fat diet; HFD + CH, High‐fat diet + coffee husks.

**FIGURE 3:**
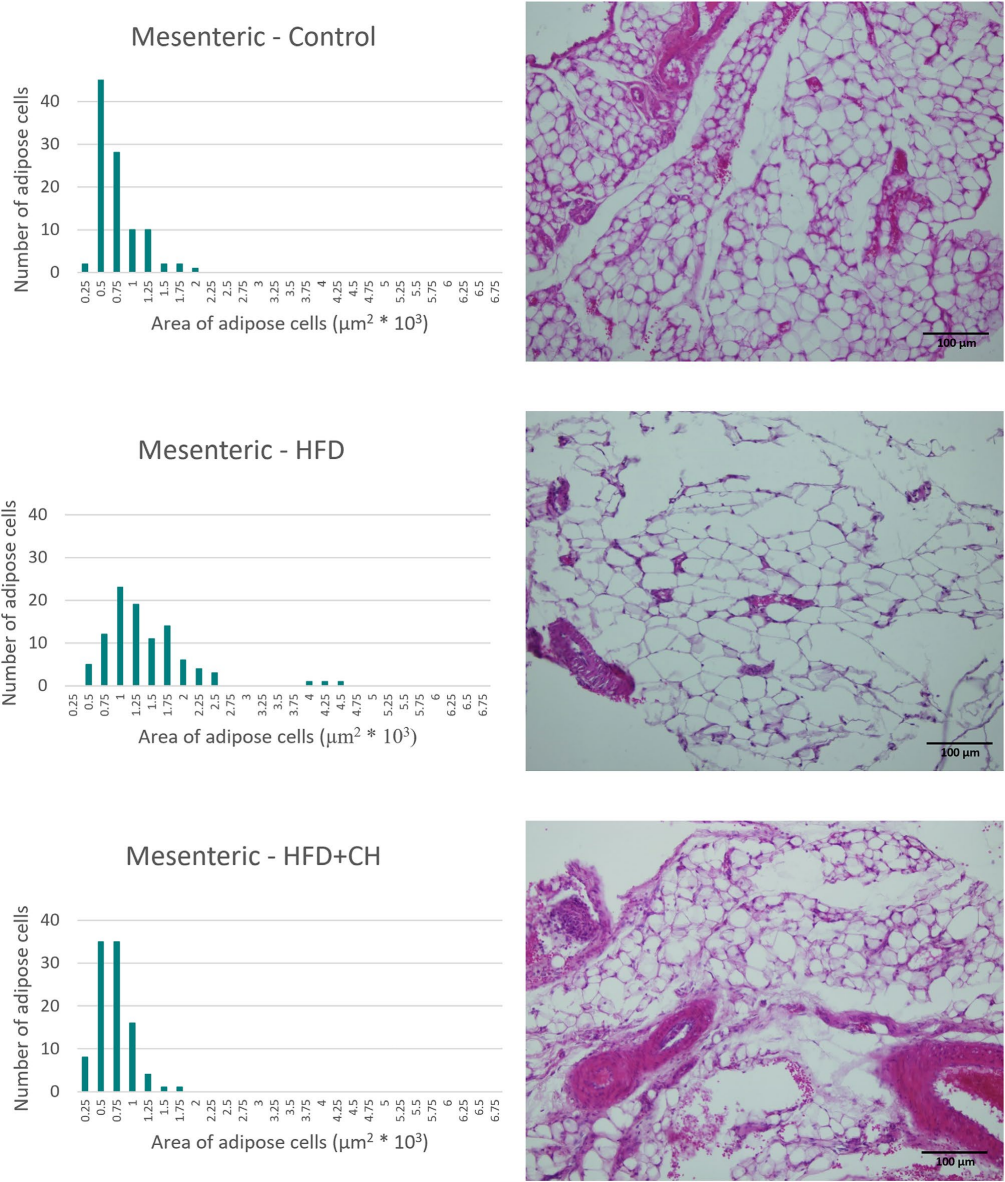
The microscopic image of adipose tissue (mesenteric) and the cell area of 100 cells in the different groups. HFD, High‐fat diet; HFD + CH, High‐fat diet + coffee husks.

**FIGURE 4:**
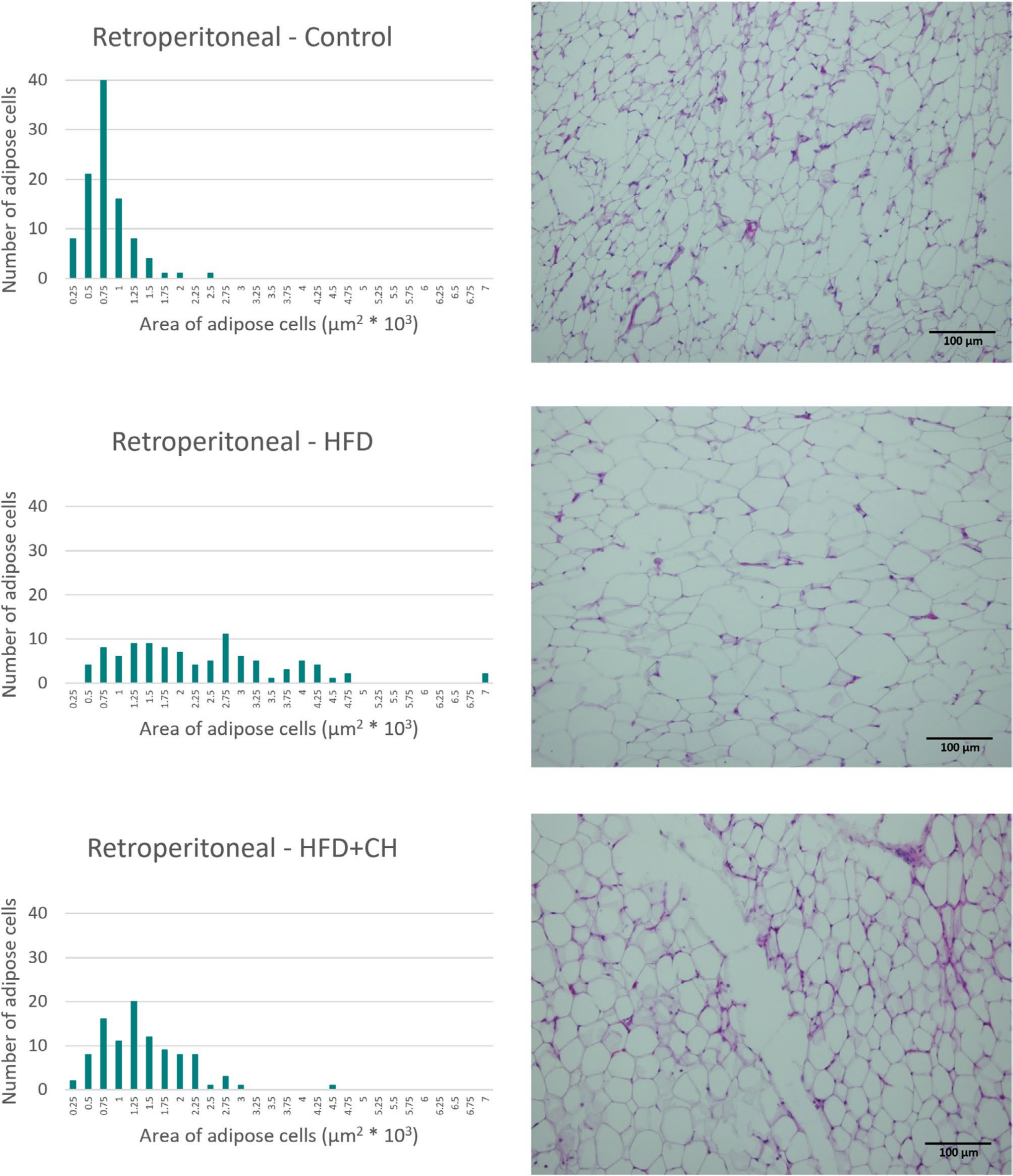
The microscopic image of adipose tissue (retroperitoneal) and the cell area of 100 cells in the different groups. HFD, high‐fat diet; HFD + CH, high‐fat diet + coffee husks.

**FIGURE 5:**
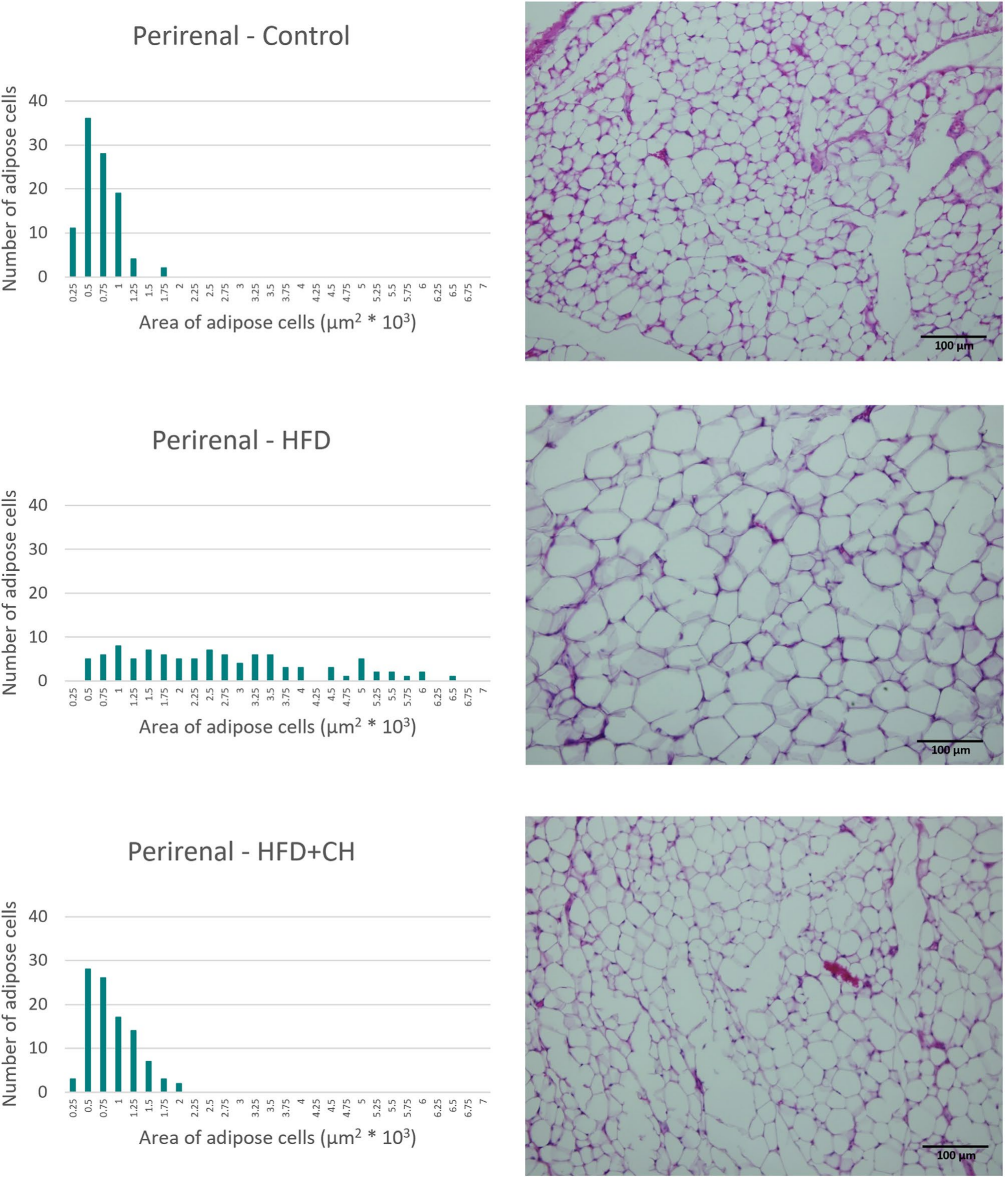
The microscopic image of adipose tissue (perirenal) and the cell area of 100 cells in the different groups. HFD, High‐fat diet; HFD + CH, High‐fat diet + coffee husks.

**FIGURE 6:**
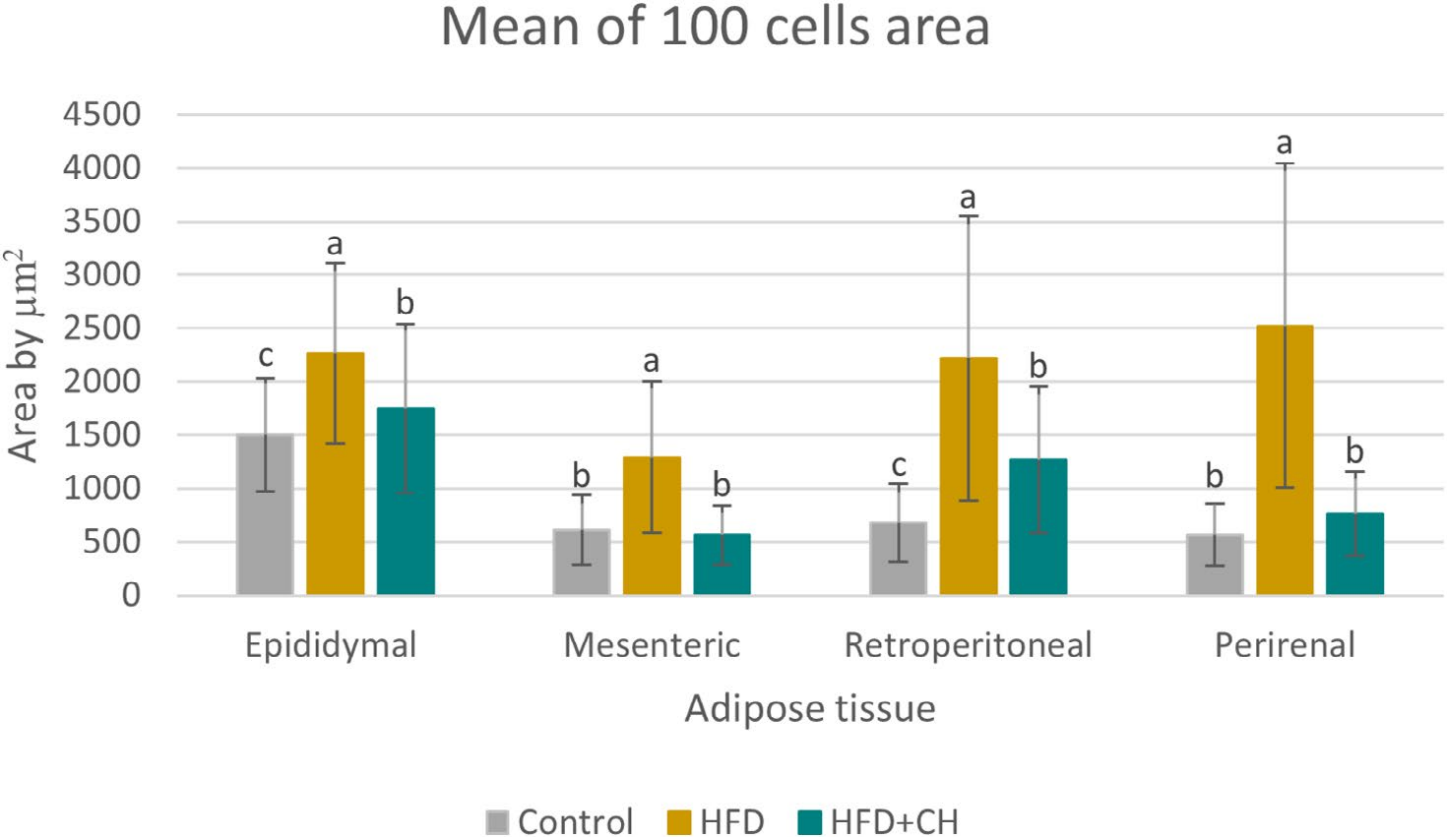
Mean of 100 cell areas (μm^2^). HFD, high‐fat diet; HFD + CH, high‐fat diet + coffee husks.

A significant increase in final weight and %BWG of the rats in the HFD group was observed compared to the control group, while the HFD + CH group had lower values with significant differences in final weight and %BWG compared to the HFD group, although they remained higher than the control group. There were no significant differences in BMI between the groups, as determined by the Welch test (F(2, 9.47) = 0.42, *p* = 0.67), and all were within the normal range for rats, ranging from 0.45 to 0.68 g/cm^2^ (Novelli et al. 2007). However, BMI is an inaccurate indicator for diagnosing obesity in animals and humans, while body fat percentage is a highly accurate indicator of obesity and overweight (Rodríguez‐Correa et al. 2020). In terms of adipose tissues, it is considered a reliable indicator for determining the development of obesity. In this study, it was observed that the HFD group had the highest percentage of total adipose tissue, epididymal, mesenteric, and retroperitoneal fat, with significant differences compared to the other groups. However, there were no significant differences between it and the HFD + CH group in the perirenal percentage. The treatment with CH contributed to reducing fat accumulation in the adipose tissues of the rats, with the ratios being close to the control group in the percentage of total adipose tissue, % epididymal, and % perirenal, while being lower than the control group in % mesenteric and % retroperitoneal. Likewise, the microscopic images, we observe that the size of cells of adipose tissues in the HFD group is larger compared to the HFD + CH group across all types of tissues. Figures 2, 3, 4, 5 show microscopic images of adipose tissues at 200× magnification and a graph illustrating the classification of 100 adipocytes by area for the same adipose tissue. From Figure 2, we find that the control group had a large number of cells (about 25 and 20 cells) with small areas (1.5 × 10^3^ and 1.25 × 10^3^ μm^2^), while the number was low in the HFD group (about 8 and 4 cells). This indicates an increase in the size of adipocytes in the epididymal tissue in the HFD group. The HFD + CH group had approximately 9 and 19 cells of the same size, respectively. It is also noted that there is a higher number of adipocytes larger than 2 × 10^3^ μm^2^ in the HFD group compared to the other groups. Figure 3 shows clear differences between the groups in the area of mesenteric adipocytes, where the total number of adipocytes with an area less than 0.75 × 10^3^ μm^2^ was very high in the control group and the HFD + CH group, with 75 and 78 cells respectively. While the total number of cells in the HFD group with an area less than 0.75 × 10^3^ μm^2^ was only 17 cells. Figure 4 also shows that the control group had the majority of small adipocytes in the retroperitoneal tissue, with very few cells having an area of 1.5 × 10^3^ μm^2^ or higher (7 cells). While the number of cells in the HFD group with an area 1.5 × 10^3^ μm^2^ or higher was 73 cells, it was 43 cells in the HFD + CH group. This may indicate the effect of coffee husks in reducing the rate of fat accumulation in adipose tissue. In Figure 5, it is shown that the number of perirenal adipocytes with an area of 2 × 10^3^ μm^2^ or greater is absent in the control group and only two in the HFD + CH group, while their number is higher in the HFD group with 62 cells. Figure 6 shows the average area of 100 adipocytes in different adipose tissues. The average area of 100 cells in the HFD group was the highest and significantly different compared to the other groups in all types of adipose tissues. In comparison between the control group and the HFD + CH group, there were no significant differences between the two groups in the average area of 100 cells for the mesenteric and perirenal tissues. The control group had the lowest average area with significant differences in the epididymal and retroperitoneal tissues. Feeding on a high‐fat diet led to a significant increase in cholesterol and triglyceride content in the liver compared to the control group. However, treatment with CH resulted in a significant reduction in cholesterol and triglyceride content in the liver compared to the HFD group.

## 4 Discussion

The coffee husks have a positive effect on blood lipids and white adipose tissues. This may be due to the presence of functional compounds in coffee husks that could influence the metabolism. Phenolic compounds such as caffeic acid and ferulic acid have a positive and beneficial effect on lipid metabolism disorders and glucose balance in experimental rodent models (Zych et al. 2019). In the Zych et al. study, they found that rats fed rosmarinic acid caused a decrease in TC and TG levels. This study's results are consistent with these findings, as the levels of TC and TG decreased in rats fed coffee husks. From Table 1, we find that coffee husks contain around 5 ppm rosmarinic acid. Protein Niemann–Pick C1‐like 1 (NPC1L1) is a key protein in the cholesterol absorption process. Caffeine competes with cholesterol and binds to this protein, leading to a decreased binding rate with cholesterol, which results in lower cholesterol levels (Sansri et al. 2024). Coffee husks contain 0.13–0.83 g of caffeine per 100 g (DePaula et al. 2025). Moreover, catechins reduce body weight and work effectively against hypercholesterolemia and hyperglycemia in Sprague Dawley rats (Ahmad et al. 2015). However, the reduction of LDL‐C and VLDL‐C in the group that consumed HFD + CH may be due to the quercetin content in CH. Quercetin reduces plasma TG and decreases liver Apob expression, which may lead to a decrease in VLDL production (Kuipers et al. 2018). Stimulating mouse liver cells with quercetin reduces the synthesis of TG and VLDL due to the decrease in ACC and diacylglycerol acyltransferase activities (Gnoni et al. 2009). Ya‐Qin reported that many phenolic compounds, such as caffeic acid, sinapic acid, and 4‐hydroxybenzoic acid, have anti‐atherosclerotic and anti‐inflammatory effects and protect the heart and blood vessels (Ma et al. 2008). 4‐Hydroxybenzoic acid, when administered orally to diabetic rats, reduced blood glucose levels by increasing glucose consumption in the blood, but it did not raise insulin levels or change glycogen content in the liver (Peungvicha et al. 1998). In this study, the blood glucose levels in the HFD + CH group were at intermediate levels between the control group and the HFD group. The high‐fat diet may lead to an increase in blood glucose levels compared to a moderate diet. When rats were fed an HFD containing CH, it led to a reduction in blood glucose levels. This decrease may be due to the presence of 4‐hydroxybenzoic acid. 4‐Hydroxybenzoic acid regulates the expression of the glucose transporter GLUT4 and has an effect on activating PPARy, which reduces blood glucose levels by enhancing glucose metabolism and the storage of glycogen in the liver (López‐Herrador et al. 2025). From Table 1, we find that coffee husks contain around 254.2 ppm. Also, 4‐hydroxybenzoic acid has a positive effect on reducing the weight of obese mice by lowering the percentage of adipose tissues in the mice (Díaz‐Casado et al. 2024). The results show that the HFD + CH group has a lower percentage of adipose tissues compared to the HFD group.

With these findings, it is important to acknowledge that this study focused on a single dose of 7% coffee husk. Further studies are needed to investigate various doses to gain a clearer understanding of the coffee husk effect on blood plasma parameters and white adipose tissues.

## 5 Conclusion

The study demonstrates that coffee husks contain valuable phenolic compounds that have bioactive properties. The results demonstrated that consuming an HFD led to an increase in plasma indicators (TC, LDL, TG, and blood glucose), reflecting its metabolic impact on rats. However, the diet supplemented with coffee husks along with a high‐fat diet showed a significant improvement in those plasma indicators. Furthermore, coffee husks contributed to reducing fat accumulation in adipose tissues and the liver, indicating a protective effect against obesity. Coffee husks have promising potential as a functional food supplement for mitigating metabolic disorders associated with HFD. Further studies are needed to explain the mechanisms of coffee husks affecting plasma indicators. Evaluating various doses of coffee husks will be beneficial to determine the appropriate dose that gives the best positive effect on the plasma indicators and adipose tissues.

## Author Contributions

**Shaheed Mohammed Alshaikhsaleh:** investigation, writing – original draft, writing – review and editing, validation, methodology, formal analysis, resources.

## Funding

The author declares the financial support was received for the research and publication of this article. This study was supported by the Deanship of Scientific Research, the Vince Presidency for Graduate Studies and Scientific Research, at King Faisal University, under project No. (KFU261028).

## Ethics Statement

The experiment was approved by the Research Ethics Committee of the Deanship of Scientific Research, King Faisal University (KFU‐REC‐2024‐MAY‐ETHICS2263). Informed consent was not applicable as the study involved animal subjects.

## Conflicts of Interest

The author declares no conflicts of interest.

## Data Availability

The data that support the findings of this study are available from the corresponding author upon reasonable request.

## References

[fsn371987-bib-0001] Abd El Malik, A. 2019. “Changes in Lipid Profile and Heart Tissues of Wistar Rats Induces by Using Monosodium Glutamate as Food Additive.” International Journal of Biochemistry & Physiology 4, no. 1: 147. 10.23880/ijbp-16000147.

[fsn371987-bib-0002] Abd El‐Gawad, I. A. , E. M. El‐Sayed , S. A. Hafez , H. M. El‐Zeini , and F. A. Saleh . 2005. “The Hypocholesterolaemic Effect of Milk Yoghurt and Soy‐Yoghurt Containing Bifidobacteria in Rats Fed on a Cholesterol‐Enriched Diet.” International Dairy Journal 15, no. 1: 37–44. 10.1016/j.idairyj.2004.06.001.

[fsn371987-bib-0003] Abreu, T. L. , M. Estévez , L. M. de Carvalho , et al. 2024. “Unveiling the Bioactivity and Bioaccessibility of Phenolic Compounds From Organic Coffee Husks Using an In Vitro Digestion Model.” Journal of the Science of Food and Agriculture 104, no. 3: 1833–1842. 10.1002/jsfa.13078. 37884474

[fsn371987-bib-0004] Afriansyah, A. , M. Setiawati , M. A. Suprayudi , and I. A. Fauzi . 2023. “Evaluation of Dietary Coffee Coffee Canephora Husk Supplementation on the Growth, Blood Chemicals, and Antioxidative Activity of Red Nile Tilapia Oreochromis sp.” Jurnal Akuakultur Indonesia 22, no. 1: 18–26. 10.19027/jai.22.1.18-26.

[fsn371987-bib-0005] Ahmad, R. S. , M. S. Butt , M. T. Sultan , et al. 2015. “Preventive Role of Green Tea Catechins From Obesity and Related Disorders Especially Hypercholesterolemia and Hyperglycemia.” Journal of Translational Medicine 13, no. 1: 1–9. 10.1186/s12967-015-0436-x. 25888764 PMC4351924

[fsn371987-bib-0006] Al Qaisi, Y. , I. Alfarrayeh , A. Alsarayreh , K. Khleifat , and N. Abu‐Nwas . 2024. “Assessment of Antioxidant Potential, Cytotoxicity, and Anticancer Activity of Methanolic Extracts From Selected Wild Medicinal Plants.” Phytomedicine Plus 4, no. 2: 100534. 10.1016/j.phyplu.2024.100534.

[fsn371987-bib-0007] Ali, M. A. , and S. Bhowmik . 2025. “Bioactive Compounds in Coffee Husk: Extraction, Functional Properties, Applications, and Sustainable Approach in Circular Economy.” RSC Sustainability 3, no. 10: 4410–4425. 10.1039/D5SU00531K.

[fsn371987-bib-0008] Alshaikhsaleh, S. M. , and S. A. Asiri . 2025. “Influence of Fatty Acid Profile of Moringa Peregrina Seeds Oil on Blood Plasma Lipids and Blood Glucose on Rats.” 10.18805/IJAR.BF-1954.

[fsn371987-bib-0009] Alshaikhsaleh, S. M. , F. A. Saleh , and M. M. Al‐Otaibi . 2025. “Effects of Camel Hump Fat, Palm Olein Oil, and Corn Oil Feed Additives on Plasma Lipids and Adipose Tissues in Rats.” Frontiers in Nutrition 12: 1–12. 10.3389/fnut.2025.1587579. PMC12042705 40308635

[fsn371987-bib-0011] Bouhlali, E. , T. Dine , C. Alem , et al. 2017. “Phytochemical Compositions and Antioxidant Capacity of Three Date ( *Phoenix dactylifera* L.) Seeds Varieties Grown in the South East Morocco.” Journal of the Saudi Society of Agricultural Sciences 16, no. 4: 350–357. 10.1016/j.jssas.2015.11.002.

[fsn371987-bib-0012] Braojos, C. , M. Rebollo‐Hernanz , S. Cañas , et al. 2024. “Coffee Pulp Simulated Digestion Enhances Its In Vitro Ability to Decrease Emulsification and Digestion of Fats, and Attenuates Lipid Accumulation in HepG2 Cell Model.” Current Research in Food Science 9: 100804. 10.1016/j.crfs.2024.100804. 39108698 PMC11301345

[fsn371987-bib-0013] Cangussu, L. B. , J. C. Melo , A. S. Franca , and L. S. Oliveira . 2021. “Chemical Characterization of Coffee Husks, a By‐Product of *coffea arabica* Production.” Food 10, no. 12: 3125. 10.3390/foods10123125. PMC8700850 34945676

[fsn371987-bib-0014] DePaula, J. , F. L. Partelli , A. M. Batista , V. Calado , and A. Farah . 2025. “Major Bioactive Compounds in Seeds, Husks, and Leaves of Selected Genotypes of *Coffea canephora* cv. Conilon From Three Consecutive Crops.” Plants 14, no. 7: 1–23. 10.3390/plants14071040. PMC11990690 40219109

[fsn371987-bib-0015] Díaz‐Casado, M. E. , P. González‐García , S. López‐Herrador , et al. 2024. “Oral β‐RA Induces Metabolic Rewiring Leading to the Rescue of Diet‐Induced Obesity.” Biochimica et Biophysica Acta, Molecular Basis of Disease 1870, no. 7: 167283. 10.1016/j.bbadis.2024.167283. 38851305

[fsn371987-bib-0016] El‐Anany, A. M. , and R. F. M. Ali . 2018. “Hypolipidemic Effect of Coffee Silver Skin in Rats Fed a High‐Fat Diet.” Food Science and Human Wellness 7, no. 4: 252–259. 10.1016/j.fshw.2018.10.005.

[fsn371987-bib-0017] El‐din, M. F. S. , and H. A. Elsawy . 2021. “Utilization of Coffee Husks to Prepare Functional Products.” Suez Canal University Journal of Food Sciences 8, no. 1: 19–28. 10.21608/scuj.2021.200308.

[fsn371987-bib-0018] Feingold, K. R. 2000. “Cholesterol Lowering Drugs.”

[fsn371987-bib-0019] Gao, J. , L. Ma , J. Yin , T. Li , Y. Yin , and Y. Chen . 2024. “Canola Oil Ameliorates Obesity by Suppressing Lipogenesis and Reprogramming the Gut Microbiota in Mice via the AMPK Pathway.” Nutrients 16, no. 19: 3379. 10.3390/nu16193379. 39408346 PMC11478415

[fsn371987-bib-0020] Gnoni, G. V. , G. Paglialonga , and L. Siculella . 2009. “Quercetin Inhibits Fatty Acid and Triacylglycerol Synthesis in Rat‐Liver Cells.” European Journal of Clinical Investigation 39, no. 9: 761–768. 10.1111/j.1365-2362.2009.02167.x. 19508303

[fsn371987-bib-0021] Gong, X. , X. Li , Y. Xia , et al. 2020. “Effects of Phytochemicals From Plant‐Based Functional Foods on Hyperlipidemia and Their Underpinning Mechanisms.” Trends in Food Science & Technology 103: 304–320. 10.1016/j.tifs.2020.07.026.

[fsn371987-bib-0022] Harini, M. , and O. P. Astirin . 1970. “Blood Cholesterol Levels of Hypercholesterolemic Rat ( *Rattus norvegicus* ) After VCO Treatment.” Nusantara Bioscience 1, no. 2: 53–58. 10.13057/nusbiosci/n010201.

[fsn371987-bib-0023] Hidayaturrahmah , H. Budi Santoso , R. Aulia Rahmi , and D. Kartikasari . 2020. “Blood Glucose Level of White Rats ( *Rattus norvegicus* ) After Giving Catfish Biscuit (*Pangasius hypothalmus*).” BIO Web of Conferences 20: 4005. 10.1051/bioconf/20202004005.

[fsn371987-bib-0024] Karam, I. , M. Yang , and J. Li . 2018. “Induce Hyperlipidemia in Rats Using High Fat Diet Investigating Blood Lipid and Histopathology.” Journal of Hematology and Blood Disorders 4: 104. 10.15744/2455-7641.4.104.

[fsn371987-bib-0048] Kirk, S. , and R. Sawyer . 1991. Pearson's Composition and Analysis of Foods. 9th ed. Longman Group Ltd.

[fsn371987-bib-0025] Kruit, J. K. , A. K. Groen , T. J. van Berkel , and F. Kuipers . 2006. “Emerging Roles of the Intestine in Control of Cholesterol Metabolism.” World Journal of Gastroenterology 12, no. 40: 6429–6439. 10.3748/wjg.v12.i40.6429. 17072974 PMC4100631

[fsn371987-bib-0026] Kuipers, E. N. , A. D. van Dam , N. M. Held , et al. 2018. “Quercetin Lowers Plasma Triglycerides Accompanied by White Adipose Tissue Browning in Diet‐Induced Obese Mice.” International Journal of Molecular Sciences 19, no. 6: 1–14. 10.3390/ijms19061786. PMC6032193 29914151

[fsn371987-bib-0027] Kusumah, J. , and E. Gonzalez de Mejia . 2022. “Coffee Constituents With Antiadipogenic and Antidiabetic Potentials: A Narrative Review.” Food and Chemical Toxicology 161: 112821. 10.1016/j.fct.2022.112821. 35032569

[fsn371987-bib-0028] Li, W. , C. Yang , X. Mei , et al. 2021. “Effect of the Polyphenol‐Rich Extract From *Allium cepa* on Hyperlipidemic Sprague‐Dawley Rats.” Journal of Food Biochemistry 45, no. 1: 1–9. 10.1111/jfbc.13565. 33219537

[fsn371987-bib-0029] López‐Herrador, S. , J. Corral‐Sarasa , P. González‐García , et al. 2025. “Natural Hydroxybenzoic and Hydroxycinnamic Acids Derivatives: Mechanisms of Action and Therapeutic Applications.” Antioxidants 14, no. 6: 1–40. 10.3390/antiox14060711. PMC12189131 40563342

[fsn371987-bib-0030] Ma, Y.‐Q. , X.‐Q. Ye , Z.‐X. Fang , J.‐C. Chen , G.‐H. Xu , and D.‐H. Liu . 2008. “Phenolic Compounds and Antioxidant Activity of Extracts From Ultrasonic Treatment of Satsuma Mandarin (*Citrus unshiu* Marc.) Peels.” Journal of Agricultural and Food Chemistry 56, no. 14: 5682–5690. 10.1021/jf072474o. 18572916

[fsn371987-bib-0031] Mahdi, C. , P. Citrawati , and V. F. Hendrawan . 2020. “The Effect of Rice Bran on Triglyceride Levels and Histopatologic Aorta in Rat ( *Rattus norvegicus* ) of High Cholesterol Dietary Model.” IOP Conference Series: Materials Science and Engineering 833, no. 1: 12022. 10.1088/1757-899X/833/1/012022.

[fsn371987-bib-0032] Moon, Y. , T. Tong , W. Kang , and T. Park . 2019. “Filbertone Ameliorates Adiposity in Mice Fed a High‐Fat Diet via Activation of cAMP Signaling.” Nutrients 11, no. 8: 1–14. 10.3390/nu11081749. PMC6723245 31366045

[fsn371987-bib-0033] Nielsen, S. S. 2019. “Correction to: Food Analysis Laboratory Manual.” In Food Analysis Laboratory Manual, C1–C2. Springer. 10.1007/978-3-319-44127-6.

[fsn371987-bib-0034] Novelli, E. L. B. , Y. S. Diniz , C. M. Galhardi , et al. 2007. “Anthropometrical Parameters and Markers of Obesity in Rats.” Laboratory Animals 41, no. 1: 111–119. 10.1258/002367707779399518. 17234057

[fsn371987-bib-0035] Ozen, E. , R. Mihaylova , M. Weech , S. Kinsella , J. A. Lovegrove , and K. G. Jackson . 2022. “Association Between Dietary Saturated Fat With Cardiovascular Disease Risk Markers and Body Composition in Healthy Adults: Findings From the Cross‐Sectional BODYCON Study.” Nutrition and Metabolism 19, no. 1: 1–15. 10.1186/s12986-022-00650-y. 35241101 PMC8896371

[fsn371987-bib-0036] Peungvicha, P. , S. Thirawarapan , and H. Watanabe . 1998. “Possible Mechanism of Hypoglycemic Effect of 4‐Hydroxybenzoic Acid, a Constituent of Pandanus Odorus Root.” Japanese Journal of Pharmacology 78: 395–398. 10.1254/jjp.78.395. 9869276

[fsn371987-bib-0037] Rodríguez‐Correa, E. , I. González‐Pérez , P. I. Clavel‐Pérez , Y. Contreras‐Vargas , and K. Carvajal . 2020. “Biochemical and Nutritional Overview of Diet‐Induced Metabolic Syndrome Models in Rats: What Is the Best Choice?” Nutrition & Diabetes 10, no. 1: 24. 10.1038/s41387-020-0127-4. 32616730 PMC7331639

[fsn371987-bib-0038] Sansri, V. , M. Sroyraya , P. Phisalprapa , et al. 2024. “Suppressive Effect of Coffee Leaves on Lipid Digestion and Absorption In Vitro.” Food 13, no. 15: 1–14. 10.3390/foods13152445. PMC11312072 39123636

[fsn371987-bib-0039] Santos, R. M. M. , and D. R. A. Lima . 2016. “Coffee Consumption, Obesity and Type 2 Diabetes: A Mini‐Review.” European Journal of Nutrition 55, no. 4: 1345–1358. 10.1007/s00394-016-1206-0. 27026242

[fsn371987-bib-0040] Silva, G. S. , M. H. G. Gomes , L. M. de Carvalho , et al. 2024. “Microencapsulation of Organic Coffee Husk Polyphenols: Effects on Release, Bioaccessibility, and Antioxidant Capacity of Phenolics in a Simulated Gastrointestinal Tract.” Food Chemistry 434: 137435. 10.1016/j.foodchem.2023.137435. 37713755

[fsn371987-bib-0041] Sirotkin, A. V. , and A. Kolesárová . 2021. “The Anti‐Obesity and Health‐Promoting Effects of Tea and Coffee.” Physiological Research 70, no. 2: 161–168. 10.33549/physiolres.934674. 33992045 PMC8820582

[fsn371987-bib-0042] Sun, P. , L. Zhao , N. Zhang , et al. 2021. “Bioactivity of Dietary Polyphenols: The Role in LDL‐C Lowering.” Food 10, no. 11: 1–29. 10.3390/foods10112666. PMC8617782 34828946

[fsn371987-bib-0043] Ward, N. C. , G. F. Watts , and R. H. Eckel . 2019. “Statin Toxicity: Mechanistic Insights and Clinical Implications.” Circulation Research 124, no. 2: 328–350. 10.1161/CIRCRESAHA.118.312782. 31170055

[fsn371987-bib-0044] Yang, C. , L. Yifan , L. Dan , Y. Qian , and J. Ming‐yan . 2015. “Bamboo Leaf Flavones and Tea Polyphenols Show a Lipid‐Lowering Effect in a Rat Model of Hyperlipidemia.” Drug Research 65, no. 12: 668–671. 10.1055/s-0035-1547253. 25970469

[fsn371987-bib-0045] Zainal Abidin, A. , H. Bahari , F. Othman , N. Norkhairani , and S. Balan . 2025. “The Effectiveness of Consuming Liberica Coffee Pulp Yoghurt on Hypercholesterolemia Induced Male Sprague Dawley Rats.” Natural and Life Sciences Communications 24: 31. 10.12982/NLSC.2025.031.

[fsn371987-bib-0046] Zhao, W. , J. Li , X. He , O. Lv , Y. Cheng , and R. Liu . 2014. “In Vitro Steatosis Hepatic Cell Model to Compare the Lipid‐Lowering Effects of Pomegranate Peel Polyphenols With Several Other Plant Polyphenols as Well as Its Related Cholesterol Efflux Mechanisms.” Toxicology Reports 1: 945–954. 10.1016/j.toxrep.2014.10.013. 28962306 PMC5598384

[fsn371987-bib-0047] Zych, M. , I. Kaczmarczyk‐Sedlak , W. Wojnar , and J. Folwarczna . 2019. “Effect of Rosmarinic Acid on the Serum Parameters of Glucose and Lipid Metabolism and Oxidative Stress in Estrogen‐Deficient Rats.” Nutrients 11, no. 2: 267. 10.3390/nu11020267. 30691017 PMC6412204

